# Genome Analysis of a Tembusu Virus, GX2013H, Isolated from a Cheery Valley Duck in Guangxi, China

**DOI:** 10.1128/genomeA.00466-14

**Published:** 2014-07-10

**Authors:** Zhixun Xie, Tingting Zeng, Liji Xie, Xianwen Deng, Zhiqin Xie, Jiabo Liu, Qing Fan, Yaoshan Pang, Sisi Luo

**Affiliations:** Guangxi Key Laboratory of Animal Vaccines and Diagnostics, Guangxi Veterinary Research Institute, Nanning, Guangxi Province, China

## Abstract

We report here the complete genome sequence of a duck Tembusu virus (DTMUV) strain, GX2013H, isolated from a duck from Cheery Valley in the Guangxi Province of southern China in 2013. We obtained the strain GX2013H from a Cheery Valley duck with severely decreased egg production and neurological signs. The genome of GX2013H is 10,990 nucleotides (nt) in length and contains a single open reading frame encoding a putative polyprotein of 3,425 amino acids (aa). A comparison of the complete sequence and the deduced amino acid sequence of GX2013H with published sequences of 15 other chicken anemia viruses from China showed that the homologies of the nucleotides are approximately 96.5% to 97.5% and the homologies of the deduced amino acid sequences are approximately 98.9% to 99.3%. This report will help to understand the epidemiology and molecular characteristics of TMUV in Guangxi.

## GENOME ANNOUNCEMENT

In 2010, a novel infectious agent emerged in China and caused extensive epidemics in layer and breeder ducks. The isolated strain was considered to be a new genotype of Tembusu virus (TMUV) (1). TMUV infects mainly ducks and geese (2, 3), and nearly all duck species can be infected by TMUV, such as Beijing ducks, Muscovy ducks, and Cheery Valley ducks (1, 2, 4, 5). There was nearly 100% morbidity but 0 to 12% mortality in infected ducks, which were observed in the area of greatest waterfowl production in China. TMUV causes a range of symptoms in infected ducks, including decreased egg production, high fever, loss of appetite, and neurological signs. To date, many TMUVs have been isolated and viral genomes have been sequenced (3, 6–9). However, genome sequences of strains isolated from Guangxi have rarely been published.

In 2013, breeder ducks showed a decrease in egg production from 90% to almost 0%, as well as neurological signs, in a Cheery Valley duck farm in Guangxi. We collected ovary and brain samples from affected ducks and isolated the virus under the conventional procedure (10). A strain of TMUV was isolated, and other pathogens that cause similar symptoms were ruled out (11). Thirteen pairs of primers were designed to amplify the different genomic regions of the strain GX2013H, with an overlapping genome fragment covering each region. The 5′- and 3′-terminal sequences were determined using the SMARTer RACE cDNA amplification kit (Clontech). The amplified products were purified, cloned into pMD-18T vector (Takara), and sequenced (Invitrogen, Shanghai, China). The sequences were assembled using the SeqMan program to produce the complete genome sequence of GX2013H (12, 13). The full-length genome sequence of GX2013H is 10,990 nucleotides (nt) in length, with a typical flavivirus genome organization, and the 5′ and 3′ untranslated regions (UTR) are 94 and 618 nt, respectively. Additionally, the coding region of GX2013H includes a single open reading frame (ORF) (10,278 nt) that encodes a polypeptide of 3,425 amino acids (aa), three structural proteins (capsid, prM, and envelope), and seven nonstructural proteins (NS1, NS2A, NS2B, NS3, NS4A, NS4B, and NS5).

Compared to the genome sequences of previously isolated TMUVs from different species of ducks and geese in various areas in China, there is 96.5% to ~97.5% homology at the nucleotide level and 98.9% to ~99.3% homology at the amino acid level. A phylogenetic tree based on the whole polyprotein sequence showed that GX2013H is in a single clade, whereas other strains are in a different clade.

By predicting the potential glycosylation sites, we found 13 glycosylation sites in six viral proteins. The numbers of glycosylation sites in the prM, E, NS1, NS2A, NS4B, and NS5 genes are 2, 1, 3, 1, 3, and 3, respectively.

In conclusion, the study of the whole-genome sequence of TMUV profits further investigation on the epidemiology and evolution of TMUV, and it may help elucidate the mechanisms of virus replication and pathogenesis.

### Nucleotide sequence accession number.

The complete genome sequence of the duck Tembusu virus isolate has been deposited to GenBank under the accession no. KJ700462.
